# Dual consent? Donors’ and recipients’ views about involvement in decision-making on the use of embryos created by gamete donation in research

**DOI:** 10.1186/s12910-019-0430-6

**Published:** 2019-12-02

**Authors:** I. Baía, C. de Freitas, C. Samorinha, V. Provoost, S. Silva

**Affiliations:** 1 EPIUnit - Instituto de Saúde Pública, Universidade do Porto, Rua das Taipas, nº 135, 4050-600 Porto, Portugal; 2 Departamento de Ciências da Saúde Pública e Forenses e Educação Médica, Faculdade de Medicina, Universidade do Porto, Porto, Portugal; 3 Centre for Research and Studies in Sociology, University Institute of Lisbon (ISCTE-IUL), Lisbon, Portugal; 4 Bioethics Institute Ghent, Department of Philosophy and Moral Sciences Ghent University, Ghent, Belgium

**Keywords:** Gamete donation, Embryo research, Consent forms, Stakeholder participation, Ethics, research

## Abstract

**Background:**

Reasonable disagreement about the role awarded to gamete donors in decision-making on the use of embryos created by gamete donation (EGDs) for research purposes emphasises the importance of considering the implementation of participatory, adaptive, and trustworthy policies and guidelines for consent procedures. However, the perspectives of gamete donors and recipients about decision-making regarding research with EGDs are still under-researched, which precludes the development of policies and guidelines informed by evidence. This study seeks to explore the views of donors and recipients about who should take part in consent processes for the use of EGDs in research.

**Methods:**

From July 2017 to June 2018, 72 gamete donors and 175 recipients completed a self-report structured questionnaire at the Portuguese Public Bank of Gametes (response rate: 76%). Agreement with dual consent was defined as the belief that the use of EGDs in research should be consented by both donors and recipients.

**Results:**

The majority of participants (74.6% of donors and 65.7% of recipients) were willing to donate embryos for research. Almost half of the donors (48.6%) and half of the recipients (46.9%) considered that a dual consent procedure is desirable. This view was more frequent among employed recipients (49.7%) than among non-employed (21.4%). Donors were less likely to believe that only recipients should be involved in giving consent for the use of EGDs in research (25.0% vs. 41.7% among recipients) and were more frequently favourable to the idea of exclusive donors’ consent (26.4% vs. 11.4% among recipients).

**Conclusions:**

Divergent views on dual consent among donors and recipients indicate the need to develop evidence-based and ethically sustainable policies and guidelines to protect well-being, autonomy and reproductive rights of both stakeholder groups. More empirical research and further theoretical normative analyses are needed to inform people-centred policy and guidelines for shared decision-making concerning the use of EGDs for research.

## Background

Guidelines, regulations, and policies on who should be involved in giving consent for the use of embryos created by gamete donation (EGDs) in research vary substantially across countries. This is especially evident regarding the role awarded to gamete donors. The European Society of Human Reproduction and Embryology and the American National Institutes of Health acknowledge that donors relinquish all rights of ownership to the gametes from the moment embryos are created [1, 2]. From this perspective, consent should concern only the recipients, as is currently the case in countries such as Portugal [3]. In contrast, the American Society for Reproductive Medicine and the Canadian Institutes of Health Research Guidelines are in favour of a dual consent, advising that donors should also have a say in decision-making about the use of embryos for research purposes when these embryos were created with their gametes [4, 5].

These heterogeneous informed consent practices on EGDs disposition are based on different meanings and interpretations attributed to traditional ethical principles and biorights [6] related to autonomy [7, 8] and ownership [7, 9]. Those who advocate for dual consent emphasize the need to acknowledge donors’ views, values, and preferences and to respect their autonomy and reproductive rights, alongside those of recipients [10–12]. In fact, the literature reveals that the availability of donated gametes for reproductive purposes does not mean that their donors consent to their use in research [2, 13]. Conversely, critical approaches that plead for consent only given by recipients encompass concerns about the protection of donors’ autonomy and with a breach of altruism, which is the primary value underlying donation [1, 14].

Reasonable disagreement about dual consent on the use of EGDs for research purposes justifies the implementation of empirically grounded, participatory, adaptive, and trustworthy policies and guidelines for consent procedures [15]. Respect for personal autonomy, i.e. for individual’s ability to make their own responsible choices knowing the available possibilities [16], is particularly difficult to translate into practice [17]. Being mindful of both donors and recipients’ preferences in the consent process can help to ensure that no one is involved in an eventually undesired procedure [12, 18]. However, the perspectives of gamete donors and recipients on decision-making regarding research with EGDs are still under-researched, which precludes the development of policies and guidelines informed by evidence. This study seeks to contribute to the debate on dual consent by drawing on an empirical study carried out in Portugal, where signing of consent forms regarding embryo donation for research purposes is only asked from recipients [3]. Similar to other European countries, Portuguese gamete donation policy is undergoing change. In 2016, entitlement to fertility treatments was extended to all women aged between 18 and 49 years, independently of sexual orientation or marital status [19]. Such transitions have increased demand for donated gametes [20] and embryo disposition is likely to follow suit in the future. This research explores the views of donors and recipients about who should take part in consent processes concerned with the use of EGDs in research.

## Methods

### Data collection and analysis

From July 2017 to June 2018, gamete donors and recipients who attended at least one medical appointment at the Portuguese Public Bank of Gametes were invited to participate in a quantitative cross-sectional study. This Bank of Gametes is located at a public hospital which performs in vitro fertilization/intracytoplasmic sperm injection in homologous and heterologous cycles. Ethical approval was granted by the Portuguese Data Protection Authority and the Ethics Committee for Health from the hospital where data was collected. Written informed consent was obtained from all donors and recipients prior to participation in the study.

At the end of the medical appointment, donors and recipients received an informative leaflet from a health professional. Subsequently, a research team member invited them to participate in the study. Those who agreed to participate were then accompanied to a private room at the Portuguese Public Bank of Gametes where they read and signed the informed consent and completed a self-report questionnaire. Of the 329 people invited, 72 donors and 179 recipients agreed to participate in the questionnaire (response rate: 76.3%).

The structured questionnaire was developed by the research team to assess ethical, legal and social issues involved in gamete donation, based on a review of literature and an exhaustive inventory of existing questionnaires on the subject. The questionnaire was validated by experts from the social and health sciences and by a pilot administration to donors and recipients. This process resulted in linguistic modifications and some items were removed. Following the fine-tuning, a final version of the questionnaire was devised encompassing a total of 34 questions divided into four sections: 1. Opinions about access to and governance of gamete donation (e.g. awareness of communication campaigns, views about preferred forms of payment to- and recruitment of- donors, anonymity); 2. Willingness to donate gametes to family and friends and for research purposes, as well as to receive gametes from family, friends or unknown donors; 3. Willingness to donate embryos for reproductive and research purposes, including views about who should be involved in decision-making on the use of EGDs in research; and 4. Sociodemographic and reproductive characteristics. A translation of the questionnaire by the authors is available as Additional file 1.

For the purposes of this paper we only analysed results regarding the views of donors and recipients about dual consent on the use of EGDs for research purposes and their association with the following sociodemographic and reproductive characteristics: sex, age, experience with gamete donation (categorized as gamete donors and recipients), marital status (categorized as married/living with the partner and single/divorced), working status (categorized as employed and other, including unemployed, students and retired), educational level, perceived income adequacy, previous experience on gamete donation and willingness to donate embryos for research. Educational level was assessed through a multiple-choice item with the following answer categories: 1) None, and can’t read or write; 2) None, but can read and write; 3) 1st cycle of basic education (4th grade); 4) 2nd cycle of basic education (6th grade); 5) 3rd cycle of basic education (9th grade); 6) Secondary education (12th grade); 7) Bachelor’s degree; 8) Licentiate degree; 9) Master’s/Integrated Master’s; 10) PhD. For analysis, this variable was dichotomized in ≤ Secondary education (12th grade) and > Secondary education (12th grade). Perceived income adequacy was assessed through the question: “Thinking of your household income, would you say that your household is able to make ends meet?”, being the answers recoded into a dichotomous variable: 1) insufficient, including respondents who reported subjective economic hardship (insufficient or caution with expenses); 2) sufficient, including respondents who reported that their household income is enough to make ends meet or comfortable. Participants were considered to have previous experience on gamete donation when recipients had at least one previous heterologous treatment or donors have donated gametes at least once before the current donation.

Willingness to donate embryos for research was assessed using a 5-point Likert scale ranging from “very unwilling” to “very willing” (range: 0–4). For this analysis, the answers were recoded into a dichotomous variable: “willing to donate” (answers scoring 3 or 4) or “other” (answers ranging between 0 and 2). The views of donors and recipients about dual consent were assessed by the question: “In your opinion, who should be involved in giving consent to the use of embryos created by gamete donation in research?”. Response options were: gamete recipients; gamete donors; both recipients and gamete donors. These options were dichotomized as “agree” (participants who answered “both recipients and gamete donors”) and “disagree” (all other participants) with dual consent. Four participants with missing values for this variable were excluded, resulting in 72 donors and 175 recipients to be included in the quantitative analysis.

### Statistical analysis

Categorical variables were presented as counts and proportions, while continuous variables were summarized as medians and percentiles. Chi-square and Mann-Whitney tests were applied, respectively, to assess the associations and mean differences between variables. Statistical significance was set at a value of *p* < 0.05. Analyses were performed using SPSS version 24.0 (Armonk, NY, USA).

## Results

Most participants were female and employed, had no children and no previous experience of gamete donation, and perceived their income as sufficient (Table 1). Donors were younger, higher educated and more frequently single or divorced (Table 2). The majority of participants (74.6% of donors and 65.7% of recipients) were willing to donate embryos for research.

**Table 1:** Opinion about involvement in consenting the use of EGDs in research, according to participants’ characteristics

	Who should be involved in giving consent to the use of EGDs in research			
	Total	Gamete recipients	Gamete donors	Both recipients and gamete donors
Overall, *n* (%)	247	91 (36.8)	39 (15.8)	117 (47.4)
Experience with donation, *n* (%)				
Donors	72	18 (25.0)*	19 (26.4)*	35 (48.6)*
Recipients	175	73 (41.7)*	20 (11.4)*	82 (46.9)*
Sex, *n* (%)				
Female	156	60 (38.5)	27 (17.3)	69 (44.2)
Male	91	31 (34.1)	12 (13.2)	48 (52.7)
Age, Median (P25-P75)	35.0	37.0 (34.0–40.0)	37.0 (33.5–40.0)	36.0 (35.0–39.0)
Educational level, *n* (%)				
≤ 12th grade	130	48 (36.9)	21 (16.2)	61 (46.9)
> 12th grade	113	40 (35.4)	18 (15.9)	55 (48.7)
Marital status, *n* (%)				
Married/Living with the partner	172	71 (41.3)*	21 (12.2)*	80 (46.5)*
Single/Divorced	74	19 (25.7)*	18 (24.3)*	37 (50.0)*
Working status, *n* (%)				
Employed	199	70 (35.2)	31 (15.6)	98 (49.2)
Other^a^	45	19 (42.2)	8 (17.8)	18 (40.0)
Perceived income adequacy, *n* (%)				
Insufficient	72	31 (43.0)	12 (16.7)	29 (40.3)
Sufficient	174	59 (33.9)	27 (15.5)	88 (50.6)
Parental status, *n* (%)				
No children	215	79 (36.8)	34 (15.8)	102 (47.4)
Children	31	11 (35.5)	5 (16.1)	15 (48.4)
Previous experience on donation, *n* (%)				
No	185	66 (35.7)	32 (17.3)	87 (47.0)
Yes	62	25 (40.3)	7 (11.3)	30 (48.4)
Embryo donation for research, *n* (%)				
Willing	162	59 (36.4)	27 (16.7)	76 (46.9)
Other^b^	75	27 (36.0)	10 (13.3)	38 (50.7)

NOTES: The total may not add up to 247 participants in each variable due to missing values

^a^Unemployed (*n* = 15), students (*n* = 29) and retired (*n* = 1); ^b^Participants who answered “very unwilling”, “unwilling” or “neither willing nor unwilling”; * *p* < 0.05

**Table 2:** Donors’ and recipients’ opinion about dual consent for using EGDs in research

	Dual consent					
	Donors			Recipients		
	Total *n* = 72	Disagree *n* = 37	Agree *n* = 35	Total *n* = 175	Disagree *n* = 93	Agree *n* = 82
Sex, *n* (%)						
Female	47	27 (57.4)	20 (42.6)	109	60 (55.0)	49 (45.0)
Male	25	10 (40.0)	15 (60.0)	66	33 (50.0)	33 (50.0)
Age, Median (P25-P75)	27.0	27.0 (25.0–31.0)	27.0 (24.0–30.0)	37.0	37.0 (34.0–40.0)	36.0 (35.0–39.0)
Educational level, *n* (%)						
≤ 12th grade	31	14 (45.2)	17 (54.8)	99	55 (55.6)	44 (44.4)
> 12th grade	41	23 (56.1)	18 (43.9)	72	35 (48.6)	37 (51.4)
Marital status, *n* (%)						
Married/Living with the partner	13	7 (53.8)	6 (46.2)	159	85 (53.5)	74 (46.5)
Single/Divorced	59	30 (50.8)	29 (49.2)	15	7 (46.7)	8 (53.3)
Working status, *n* (%)						
Employed	40	21 (52.5)	19 (47.5)	159	80 (50.3)*	79 (49.7)*
Other^a^	31	16 (51.6)	15 (48.4)	14	11 (78.6)*	3 (21.4)*
Perceived income adequacy, *n* (%)						
Insufficient	22	12 (54.5)	10 (45.5)	50	31 (62.0)	19 (38.0)
Sufficient	50	25 (50.0)	25 (50.0)	124	61 (49.2)	63 (50.8)
Parental status, *n* (%)						
No children	58	27 (46.6)	31 (53.4)	157	86 (54.8)	71 (45.2)
Children	14	10 (71.4)	4 (28.6)	17	6 (35.3)	11 (64.7)
Previous experience on donation, *n* (%)						
No	66	35 (53.0)	31 (47.0)	119	63 (52.9)	56 (47.1)
Yes	6	2 (33.3)	4 (66.7)	56	30 (53.6)	26 (46.4)
Embryo donation for research, *n* (%)						
Willing	53	27 (50.9)	26 (49.1)	109	59 (54.1)	50 (45.9)
Other^b^	18	9 (50.0)	9 (50.0)	57	28 (49.1)	29 (50.9)

NOTES: The total may not add up to 72 donors and 175 recipients in each variable due to missing values

^a^Unemployed (*n* = 15), students (*n* = 29) and retired (*n* = 1); ^b^Participants who answered “very unwilling”, “unwilling” or “neither willing nor unwilling”; * *p* < 0.05

Donors were less likely to believe that only recipients should be involved in giving consent for the use of EGDs in research (25.0% vs. 41.7% among recipients), and were more frequently favourable to the idea of exclusive donors’ consent (26.4% vs. 11.4% among recipients) (Table 1). Participants who were married or lived with the partner more often thought that consent should concern recipients alone compared to single or divorced participants.

Almost half of the donors (48.6%) and half of the recipients (46.9%) agreed with dual consent. This view was more frequent among employed recipients (49.7% vs. 21.4% among recipients with other working status) (Table 2).

## Discussion

This study revealed that almost half of the donors and half of the recipients considered that a dual consent procedure is desirable. However, recipients and donors had divergent views about who should be involved in the process of giving consent for the use of EGDs in research. These findings point to consent procedures becoming potentially disagreeable and distressing [21] and indicate the need to develop evidence-based and ethically sustainable policies and guidelines [15] to protect the well-being, autonomy and reproductive rights of both stakeholder groups [8, 22].

Donors may feel attached to embryos created with their gametes [23–25]. Such feelings of biological connectedness may entrench a sense of ownership over donated gametes [24, 26]. From a perspective of property rights, donors own all parts of their bodies and, therefore, should be awarded the authority to decide on the destiny of their gametes for both reproductive or research purposes [27]. Transferring more control over decision-making about EGDs disposition to donors will allow policy-makers and regulators to demonstrate their appreciation for gamete donors, not least by respecting and accommodating their preferences [18].

Consent procedures for EGDs disposition that fail to involve one or more stakeholder groups risk leaving rightful parties feeling marginalised and mistrustful of gamete donation policies and guidelines. Taking donors’ and recipients’ views into consideration is thus crucial to develop people-centred policies and guidelines for consent on EGDs disposition that are mindful of all stakeholders’ values and needs, and that aim to promote ever-evolving and trusted health systems [28–30].

The collection of data at a single reproductive centre is a limitation of this study. However, this is the only Public Bank of Gametes in Portugal, which allows for the generalizability of the opinions of both donors and recipients using the public healthcare system in the country where the study was undertaken. The findings of this study can be used as a baseline for subsequent qualitative studies aiming to provide a deeper understanding of the intricacies of negotiations on consent between gamete donors and recipients in cases where there is discordance. Nevertheless, there is a need for further quantitative and qualitative assessments of the views of donors and recipients on the use of EGDs in research in other countries and settings to enable comparison and inform the development of both local and global policy for an increasingly transnational practice as is the donation of gametes.

## Conclusions

This study uncovers a need to promote an open discussion about ethically sustainable consent procedures in the field of gamete donation. It also stresses the importance of conducting more empirical research and further theoretical normative analyses [21] to inform people-centred policy and guidelines for shared decision-making concerning the use of EGDs for research.

## Supplementary information

**Additional file 1.** “Gamete Donation: Public involvement and people-centred care Questionnaire”. A translation of the questionnaire developed by the research team to assess ethical, legal and social issues involved in gamete donation.

## Data Availability

The datasets generated and analysed during the current study are not publicly available due to a confidentiality agreement securing participants’ privacy and anonymity but are available from the corresponding author on reasonable request.

## Abbreviation

EGDs: Embryos created by gamete donation

## References

[1] European Society of Human Reproduction and Embryology (ESHRE) Task Force on Ethics and Law. II (2001). The cryopreservation of human embryos. Hum Reprod.

[2] Lo B, Parham L, Cedars M, Fisher S, Gates E, Giudice L (2010). Research ethics. NIH guidelines for stem cell research and gamete donors. Science..

[3] de Portugal G. Lei n.° 32/2006 de 26 de julho. Procriação Medicamente Assistida. Portugal: Diário da República, 1ª Série - N°143; 2006. p. 5245–50.

[4] The Ethics Committee of American Society for Reproductive Medicine (ASRM) (2014). Informed consent and the use of gametes and embryos for research: a committee opinion. Fertil Steril.

[5] Nelson E, Mykitiuk R, Nisker J (2008). Informed consent to donate embryos for research purposes. J Obstet Gynaecol Can.

[6] Caulfield T, Murdoch B (2017). Genes, cells, and biobanks: yes, there's still a consent problem. PLoS Biol.

[7] Archard D (2008). Informed consent: autonomy and self-ownership. J Appl Philos.

[8] Buyx A, Del Savio L, Prainsack B, Volzke H (2017). Every participant is a PI. Citizen science and participatory governance in population studies. Int J Epidemiol.

[9] Björkman B, Hansson SO (2006). Bodily rights and property rights. J Med Ethics.

[10] Pennings G (2002). The validity of contracts to dispose of frozen embryos. J Med Ethics.

[11] Kaye J, Whitley EA, Lund D, Morrison M, Teare H, Melham K (2015). Dynamic consent: a patient interface for twenty-first century research networks. Eur J Hum Genet.

[12] Klitzman R (2017). Unconventional combinations of prospective parents: ethical challenges faced by IVF providers. BMC Med Ethics.

[13] Lo B, Chou V, Cedars MI, Gates E, Taylor RN, Wagner RM (2003). Medicine. Consent from donors for embryo and stem cell research. Science.

[14] Pennings G, Ravel C, Girard JM, Domin-Bernhard M, Provoost V (2018). Attitude towards reciprocity as a motive for oocyte donation. Eur J Obstet Gynecol Reprod Biol.

[15] Stroud K, O'Doherty KC (2015). Ethically sustainable governance in the biobanking of eggs and embryos for research. Monash Bioeth Rev.

[16] Nuffield Council on Bioethics (2007). Public Health: ethical issues.

[17] Førde R (2012). How can empirical ethics improve medical practice?. Camb Q Healthc Ethics.

[18] Schaefer GO, Sinaii N, Grady C (2012). Informing egg donors of the potential for embryonic research: a survey of consent forms from US IVF clinics. Fertil Steril.

[19] International Federation of Fertility Societies (IFFS) (2019). IFFS surveillance 2019: global trends in reproductive policy and practice, 8th edition. Glob Reprod Health.

[20] Costa R. PMA: "É quase inevitável que as listas de espera aumentem". TSF Online. 2017; http://www.tsf.pt/sociedade/saude/interior/pma-equase-inevitavel-que-as-listas-de-espera. Accessed 5 Aug 2019.

[21] Ives J, Dunn M, Molewijk B, Schildmann J, Baeroe K, Frith L (2018). Standards of practice in empirical bioethics research: towards a consensus. BMC Med Ethics.

[22] Waldby C, Kerridge I, Boulos M, Carroll K (2013). From altruism to monetisation: Australian women's ideas about money, ethics and research eggs. Soc Sci Med.

[23] Baylis F, Widdows H (2015). Human embryos and eggs: from long-term storage to biobanking. Monash Bioeth Rev.

[24] Franklin S, Johnson CH, Jussen B, Sabean DW, Teuscher S (2013). From blood to genes?: rethinking Cosanguinity in the context of Geneticization. Blood and kinship: matter for metaphor from ancient Rome to the present.

[25] Samorinha C, Severo M, Machado H, Figueiredo B, De Freitas C, Silva S (2016). Couples' willingness to donate embryos for research: a longitudinal study. Acta Obstet Gynecol Scand.

[26] Kirkman M, Bourne K, Fisher J, Johnson L, Hammarberg K (2014). Gamete donors' expectations and experiences of contact with their donor offspring. Hum Reprod.

[27] Zeiler K (2014). Neither property right nor heroic gift, neither sacrifice nor aporia: the benefit of the theoretical lens of sharing in donation ethics. Med Health Care Philos.

[28] World Health Organisation (WHO). Framework on Integrated People-Centred Health Services Geneva: WHO Framework on IPCHS platform. http://www.who.int/servicedeliverysafety/areas/people-centred-care/en/ (2016). Accessed 17 Dec 2018.

[29] Cascio MA, Racine E (2018). Person-oriented research ethics: integrating relational and everyday ethics in research. Account Res.

[30] Samorinha C, Silva S (2016). A patient-centred approach to embryo donation for research. Isr J Health Policy Res.

