# Supplementary material for: Dual consent? Donors’ and recipients’ views about involvement in decision-making on the use of embryos created by gamete donation in research
Source: BMC Med Ethics. 2019 Dec 2;20:90. doi: 10.1186/s12910-019-0430-6 (PMC6889541; doi:10.1186/s12910-019-0430-6)
Supplement: Supplementary file 1 — Additional file 1. “Gamete Donation: Public involvement and people-centred care Questionnaire”. A translation of the questionnaire developed by the research team to assess ethical, legal and social issues involved in gamete donation. [file 12910_2019_430_MOESM1_ESM.pdf]

ID

QUESTIONNAIRE

Gamete donation: Public involvement and people-centred care

(POCI-01-0145-FEDER-016762; Ref. FCT PTDC/IVC-ESCT/6294/2014)

This study aims to assess the opinions of donors, recipients and health professionals regarding policy and healthcare provision in the field of gamete donation.

There are no right or wrong answers. We are interested in knowing your honest opinion. Thank you in advance for your collaboration and for taking the time to answer this questionnaire.

Date: |___|___| / |___|___| / |___|___|___|___|
(day) (month) (year)

GROUP I. OPINIONS ABOUT ACCESS AND GOVERNANCE

1. Please state the <u>two</u> main <u>sources</u> where do you seek information on gamete donation:

Health professionals	☐1	Newspapers	☐6
Scientific papers	☐2	Radio	☐7
Internet	☐3	Academic/professional training (graduation, masters, etc.)	☐8
Family, friends or colleagues	☐4	Other.	☐9
Television	☐5	Which?________________________	

1.1. How do you feel about the information you have on gamete donation?

Not informed at all ☐0 ☐1 ☐2 ☐3 ☐4 Highly informed

2. Are you aware of any communication campaign about gamete donation?

Yes ☐1 No ☐0 **(please go to question 3)**

2.1. What do you remember about those communication campaigns? (e.g. where did you see them; their content)

____

____

____

3. Have you ever had a <u>previous</u> experience as gamete donor?

- No ☐0
- Yes, once ☐1
- Yes, several times ☐2

4. Have you ever had a <u>previous</u> experience as gamete recipient (i.e. received a treatment with donated gametes)?

- No ☐0
- Yes, once ☐1
- Yes, several times ☐2

5. <u>Currently</u>, what is your experience with gamete donation?

- I am a donor ☐1
- I am a member of a couple who will be recipient of:
  Donated oocytes ☐2 Donated sperm ☐3 Donated embryos ☐4
- I am a female recipient, but I do not belong to a couple ☐5
- I am a health professional:
  Medical doctor ☐6 Nurse ☐7 Embryologist ☐8 Biologist ☐9 Psychologist ☐10
- Other. ☐11
  Which? ____
- Currently, I do not have any experience with gamete donation ☐12

6. Where did this/those experience/s occur?

6.1. Location of the fertility centre (you can choose <u>more than one</u> option):

Portugal ☐1 Abroad ☐2

6.2. Type of fertility centre (you can choose <u>more than one</u> option):

Public ☐1 Private ☐2

6.3. Number of centres:

One ☐1 Two ☐2 Three or more ☐3

7. There are different ways to pay people who donate oocytes and sperm. **In your opinion, which of the following proposals is the most appropriate to pay gamete donors?** (choose <u>only one</u> option)

- A fixed amount of money that is the same for all oocyte and sperm donors ☐1
- A variable amount of money according to the type of gametes donated (oocytes/sperm) ☐2
- A variable amount of money according to the donor's actual expenses or losses resulting from the donation ☐3
- A variable amount of money according to the characteristics of the donor ☐4

7.1. It is important for us to understand the reason(s) for your answer above. **Please give a brief explanation.**

____

____

____

8. There are differences in the number of donations that men and women can make throughout their lives.

8.1. In your opinion, **what is the maximum number of donations each woman should be able to make?____**

8.2. In your opinion, **what is the maximum number of donations each man should be able to make? ____**

9. In Portugal, gamete donation occurs under an anonymous regime, but there are other countries where this does not happen. **What is your opinion about donors' anonymity?**

____

____

____

10. When the resources are scarce, different criteria can be used to define priority of access to fertility treatments. **To what extent do you agree with the following sentences about priority of access to treatments with gamete donation within the National Health Service?**

**Priority of access to treatments with gamete donation should be given to…**	Strongly disagree	Disagree	Neutral	Agree	Strongly agree
Heterosexual couples, compared to same-sex couples	☐1	☐2	☐3	☐4	☐5
Married women, compared to single women	☐1	☐2	☐3	☐4	☐5
People with "normal" weight, compared to those who are overweight/obese	☐1	☐2	☐3	☐4	☐5

11. Do you think that donors should have the opportunity to select the features of the people who will receive their gametes?

Yes ☐1 No ☐0 **(please go to question 12)**

11.1. What features could they select? **(you can choose more than one option)**

- Age ☐1
- Marital status ☐2
- Sexual orientation ☐3
- Educational level ☐4
- Height and weight ☐5
- Other: ☐6

  Which?____

12. Do you think that recipients should have the opportunity to select the features of their gamete donors?

Yes ☐1 No ☐0 **(please go to question 13)**

12.1. What features could they select? **(you can choose more than one option)**

- Age ☐1
- Marital status ☐2
- Sexual orientation ☐3
- Educational level ☐4
- Height and weight ☐5
- Other: ☐6

  Which?____

13. In your opinion, **recipients should have access to**:

	No	Yes	Maybe
- Medical information about donors (e.g. blood type)	☐0	☐1	☐2
- Non-medical information about donors, excluding identification (e.g. educational level)	☐0	☐1	☐2
- Personal data about donors, including identification	☐0	☐1	☐2
- Other:	☐0	☐1	☐2

Which?____

14. In your opinion, **donors should have access to**:

	No	Yes	Maybe
- Information on whether any pregnancy have resulted from their donation	☐0	☐1	☐2
- Information on whether any children have resulted from their donation	☐0	☐1	☐2
- Medical information about recipients (e.g. blood type)	☐0	☐1	☐2
- Non-medical information about recipients, excluding identification (e.g. educational level)	☐0	☐1	☐2
- Personal data about recipients, including identification	☐0	☐1	☐2
- Medical information about children born through their donation	☐0	☐1	☐2
- Personal data about children born through their donation, including identification	☐0	☐1	☐2
- Other:	☐0	☐1	☐2

Which?____

15. There are several strategies for recruiting gamete donors. **Please point out the two strategies that you think would be most appropriate for Portugal:**

	1st	2nd
- Develop campaigns with University students	☐1	☐1
- Promote campaigns for the general population	☐2	☐2
- Increase the amount of payment to donors	☐3	☐3
- Promote donation by family members/friends of the recipients	☐4	☐4
- Promote donation by people involved in fertility treatments	☐5	☐5
- Change legislation so donors can be identified	☐6	☐6

15.1. It is important for us to understand the reason(s) for your answer above. **Please give a brief explanation.**

____

____

____

16. Currently, Portugal has a Public Bank of Gametes in Porto and two public centres that collect oocytes and sperm in Coimbra and Lisbon. **To what extent do you agree with this location of the public centres?**

Strongly disagree	Disagree	Neutral	Agree	Strongly agree
☐1	☐2	☐3	☐4	☐5

16.1. It is important for us to understand the reason(s) for your answer above. **Please give a brief explanation.**

____

____

____

GROUP II. WILLINGNESS TO DONATE AND TO RECEIVE GAMETES

17. If you had all necessary conditions to donate gametes, how willing would you be to:

	Very unwilling				Very willing
- Donate gametes for family	☐0	☐1	☐2	☐3	☐4
- Donate gametes for friends	☐0	☐1	☐2	☐3	☐4
- Donate gametes for unknown recipients	☐0	☐1	☐2	☐3	☐4
- Donate gametes for scientific research	☐0	☐1	☐2	☐3	☐4

17.1. It is important for us to understand the reason(s) for your answers above. **Please give a brief explanation.**

____

____

17.2. How willing would you be to donate gametes for:

	Very unwilling				Very willing
- Non-profit research	☐0	☐1	☐2	☐3	☐4
- Profit research	☐0	☐1	☐2	☐3	☐4

17.3. If your gametes were used for research purposes, would you like to be informed about it?

- Yes, I would like to be always informed about the use of my gametes ☐1
- Yes, I would like to be informed sometimes about the use of my gametes ☐2
- No, I would not like to be informed each time that my gametes are used ☐3
- I don't know ☐4

18. Would you advise a relative or a close friend to do a treatment with donated sperm:

	**No**	**Yes**	**Maybe**
- By a brother	☐0	☐1	☐2
- By another close relative	☐0	☐1	☐2
- By a friend	☐0	☐1	☐2
- By an unknown donor	☐0	☐1	☐2

19. Would you advise a relative or a friend to do a treatment with donated oocytes:

	**No**	**Yes**	**Maybe**
- By a sister	☐0	☐1	☐2
- By another close relative	☐0	☐1	☐2
- By a friend	☐0	☐1	☐2
- By an unknown donor	☐0	☐1	☐2

GROUP III. WILLINGNESS TO DONATE EMBRYOS

20. Imagine that you were a recipient of donated gametes and that cryopreserved embryos remained from that treatment. **How willing would you be to:**

	Very Unwilling				Very willing
- Donate embryos for other couples	☐0	☐1	☐2	☐3	☐4
- Donate embryos for scientific research	☐0	☐1	☐2	☐3	☐4

21. The cryopreserved embryos that remain from gamete donation treatments can be donated for scientific research. **In your opinion, who should be involved in giving consent to the use of embryos created by gamete donation in research?**

- Gamete recipients ☐1
- Gamete donors ☐2
- Both recipients and donors ☐3

22. On a scale from 1 to 5, in which 1 corresponds to “Strongly disagree” and 5 to “Strongly agree”, **to what extent do you agree with the following statements about biomedical research?**

	Strongly disagree	Disagree	Neutral	Agree	Strongly agree
1. I have a positive view about medical research in general	☐1	☐2	☐3	☐4	☐5
2. Medical researchers are mainly motivated by personal gain	☐1	☐2	☐3	☐4	☐5
3. Medical researchers can be trusted to protect the interests of people who take part in their studies	☐1	☐2	☐3	☐4	☐5
4. We all have some responsibility to help others by volunteering for medical research	☐1	☐2	☐3	☐4	☐5
5. Modern science does more harm than good	☐1	☐2	☐3	☐4	☐5
6. Society needs to devote more resources to medical research	☐1	☐2	☐3	☐4	☐5
7. Medical research needs to be closely regulated in order to prevent harm to research participants	☐1	☐2	☐3	☐4	☐5
8. Participating in medical research is generally safe	☐1	☐2	☐3	☐4	☐5
9. If I volunteer for medical research, I know my personal information will be kept private and confidential	☐1	☐2	☐3	☐4	☐5
10. A lot of emphasis on medical research and scientific progress is likely to harm research volunteers	☐1	☐2	☐3	☐4	☐5
11. Medical research will find cures for many major diseases during my lifetime	☐1	☐2	☐3	☐4	☐5

GROUP IV. SOCIODEMOGRAPHIC CHARACTERISTICS

23. Sex:

Female	☐1
Male	☐2

24. Year of birth: |___|___|___|___|

25. Country of origin:

Portugal	☐1
Other country	☐2

Which?__________________________________

26. Where do you currently live? Municipality: ____

27. What is your marital status?

Single	☐1	Widow/widower	☐4
Married	☐2	Divorced	☐5
Living with a partner	☐3	Separated (married, but do not live with the partner)	☐6

28. What is the highest educational level you have completed?

None, and cannot read or write	☐1	Secondary education (12th grade)	☐6
None, but can read and write	☐2	Bachelor's degree	☐7
1st cycle of basic education (4th grade)	☐3	Licentiate degree	☐8
2nd cycle of basic education (6th grade)	☐4	Master's/Integrated Master's	☐9
3rd cycle of basic education (9th grade)	☐5	PhD	☐10

29. At this moment, what is your main occupation? **(please choose only one option)**

Full-time employee	☐1	Retired	☐5
Part-time employee	☐2	Doing housework	☐6
Unemployed	☐3	Other:	☐7
Student/At school/Professional qualification	☐4	Which?____________________________________	

30. What is your current job? **(if in the previous question you answered retired, doing housework or other, please consider your last job)**

____

____

31. Thinking of your household income, would you say that your household is able to make ends meet?

Insufficient	☐1
Caution with expenses	☐2
Enough to make ends meet	☐3
Comfortable	☐4

32. Some people consider that the Portuguese society is divided into social classes**. From the following social classes, in which would you include yourself?**

Low social class ☐1

Middle-low social class ☐2

Middle-high social class ☐3

High social class ☐4

None of the above ☐5

Prefer not to answer ☐6

33. Do you have children?

No ☐0 **(please go to question 34)**

Yes ☐1

33.1. How many children do you have? ____________________

34. Have you ever been diagnosed with infertility?

No ☐0

Yes ☐1

Thank you for your collaboration!

ISPUP
INSTITUTO DE SAÚDE PÚBLICA DA UNIVERSIDADE DO PORTO

ID ☐☐☐☐☐

QUESTIONÁRIO

Doação de gâmetas: Envolvimento público e cuidados centrados nas pessoas

Este estudo pretende conhecer as opiniões de dadores, beneficiários e profissionais de saúde quanto às políticas e aos cuidados de saúde que enquadram a doação de gâmetas.

Não há respostas certas nem erradas, o que nos interessa é conhecer a sua opinião sincera. Desde já agradecemos a sua colaboração e o tempo que irá disponibilizar a responder a este questionário.

Data de preenchimento: |___|___| / |___|___| / |___|___|___|___|
(dia) (mês) (ano)

GRUPO I. OPINIÕES SOBRE ACESSO E GOVERNAÇÃO

1. Indique, por favor, quais são as duas principais fontes onde procura informação sobre doação de gâmetas?

Profissionais de saúde ☐1
Artigos científicos ☐2
Internet ☐3
Familiares, amigos ou colegas ☐4
Televisão ☐5
Jornais ☐6
Rádio ☐7
Formação académica (licenciatura, mestrado, etc.) ☐8
Outra. ☐9
Qual?___________________________________

1.1. Como se sente em relação à informação que tem sobre doação de gâmetas?

Nada informado — Muito informado

☐0 ☐1 ☐2 ☐3 ☐4

2. Recorda-se de ter visto alguma campanha ou anúncio sobre doação de gâmetas?

Sim ☐1 Não ☐0 **(p.f. passe para a pergunta 3)**

2.1. Diga-nos, por favor, do que se recorda sobre essa campanha ou anúncio. (por ex.: onde viu e o conteúdo)

____

____

____

3. Já teve alguma experiência anterior como dador/a de gâmetas?

- Não ☐0
- Sim, uma vez ☐1
- Sim, várias vezes ☐2

4. Já teve alguma experiência anterior como beneficiário/a de gâmetas (ou seja, já beneficiou de um tratamento com doação de gâmetas)?

- Não ☐0
- Sim, uma vez ☐1
- Sim, várias vezes ☐2

5. Neste momento, qual a sua relação com a doação de gâmetas?

- Sou dador/a ☐1
- Sou membro de um casal recetor/beneficiário de:
  Ovócitos doados ☐2 Espermatozoides doados ☐3 Embriões doados ☐4
- Sou recetora/beneficiária, mas não pertenço a um casal ☐5
- Sou profissional de saúde:
  Médico/a ☐6 Enfermeiro/a ☐7 Embriologista ☐8 Biólogo/a ☐9 Psicólogo/a ☐10
- Outra. ☐11
  Qual? ____
- Não tenho qualquer relação com a doação de gâmetas, neste momento ☐12

6. Onde ocorreu/ocorreram essa/s experiência/s?

6.1. Localização do centro de fertilidade **(pode assinalar mais do que uma opção):**

Portugal ☐1 Fora de Portugal ☐2

6.2. Tipo de centro de fertilidade **(pode assinalar mais do que uma opção):**

Público ☐1 Privado ☐2

6.3. Número de centros:

Um ☐1 Dois ☐2 Três ou mais ☐3

7. Existem várias formas de compensar financeiramente as pessoas que doam óvulos e espermatozoides. **Na sua opinião, qual das seguintes propostas de compensação é a mais adequada? (assinale apenas uma opção)**

- Valor monetário igual para dadores de espermatozoides e dadoras de óvulos ☐1
- Valor monetário variável de acordo com o tipo de doação (óvulos/espermatozoides) ☐2
- Valor monetário variável de acordo com as despesas efetuadas ou prejuízos resultantes da dádiva ☐3
- Valor monetário variável de acordo com as características do/a dador/a ☐4

7.1. É importante, para nós, compreender melhor a sua resposta. **Explique-nos, por favor, a sua opinião sobre o valor da compensação financeira atribuída a dadores.**

____

____

____

8. Existem diferenças no número de doações que homens e mulheres podem fazer ao longo da vida.

8.1. Na sua opinião, **qual o número máximo de doações que cada mulher deve poder fazer?** __________

8.2. Na sua opinião, **qual o número máximo de doações que cada homem deve poder fazer?** __________

9. Em Portugal, a doação é feita em regime de anonimato, mas há países em que isso não acontece. **Qual é a sua opinião sobre o anonimato dos dadores?**

____

____

____

10. Quando os recursos são escassos, podem ser utilizados critérios para indicar as pessoas que têm acesso prioritário a tratamentos. **Em que medida concorda com as seguintes afirmações sobre o acesso prioritário a tratamentos com doação de gâmetas no Serviço Nacional de Saúde?**

**Deverão ter prioridade de acesso a tratamentos com doação de gâmetas…**	Discordo totalmente	Discordo um pouco	Não concordo nem discordo	Concordo um pouco	Concordo totalmente
Os casais heterossexuais, por comparação com os homossexuais	☐1	☐2	☐3	☐4	☐5
As mulheres casadas, por comparação com as solteiras	☐1	☐2	☐3	☐4	☐5
As pessoas com um peso “normal”, por comparação com as que têm excesso de peso/obesidade	☐1	☐2	☐3	☐4	☐5

11. Considera que o/a dador/a deve ter a possibilidade de escolher características das pessoas que vão receber os seus gâmetas?

Sim ☐1 Não ☐0 **(pf. passe para a pergunta 12)**

11.1. Que características poderiam escolher? **(pode selecionar mais do que uma opção)**

- Idade ☐1
- Estado civil ☐2
- Orientação sexual ☐3
- Nível de escolaridade ☐4
- Altura e peso ☐5
- Outra: ☐6

Qual?____

12. Considera que os beneficiários devem ter a possibilidade de escolher características dos dadores de gâmetas?

Sim ☐1 Não ☐0 **(pf. passe para a pergunta 13)**

12.1. Que características poderiam escolher? **(pode selecionar mais do que uma opção)**

- Idade ☐1
- Estado civil ☐2
- Orientação sexual ☐3
- Nível de escolaridade ☐4
- Altura e peso ☐5
- Outra: ☐6

Qual?____

13. Na sua opinião, as **pessoas que beneficiam de tratamentos com doação de gâmetas <u>devem ter acesso</u>** a:

	**Não**	**Sim**	**Talvez**
- Informação médica sobre dadores (por exemplo, grupo sanguíneo)	☐0	☐1	☐2
- Informação não médica sobre dadores (por exemplo, nível de escolaridade), mas não a sua identificação	☐0	☐1	☐2
- Dados pessoais sobre os dadores, incluindo a sua identificação	☐0	☐1	☐2
- Outra:	☐0	☐1	☐2

Qual?____

14. Na sua opinião, as **pessoas que doam gâmetas <u>devem ser informadas</u>** sobre:

	**Não**	**Sim**	**Talvez**
- Se a doação resultou, ou não, numa gravidez	☐0	☐1	☐2
- Se a doação resultou no nascimento de crianças	☐0	☐1	☐2
- Dados médicos dos beneficiários (por exemplo, grupo sanguíneo)	☐0	☐1	☐2
- Dados não médicos dos beneficiários (por exemplo, nível de escolaridade), mas não a sua identificação	☐0	☐1	☐2
- Dados pessoais sobre os beneficiários, incluindo a sua identificação	☐0	☐1	☐2
- Dados médicos das crianças concebidas através da doação de gâmetas	☐0	☐1	☐2
- Dados pessoais das crianças concebidas através da doação de gâmetas, incluindo a sua identificação	☐0	☐1	☐2
- Outra:	☐0	☐1	☐2

Qual?____

15. Existem diversas estratégias para recrutar dadores de gâmetas. **Por favor, assinale as <u>duas estratégias</u> que, na sua opinião, seriam mais adequadas para Portugal:**

	1.ª	2.ª
- Desenvolver campanhas junto de estudantes universitários	☐1	☐1
- Promover campanhas para a população em geral	☐2	☐2
- Aumentar o valor da compensação financeira de dadores	☐3	☐3
- Promover a doação por parte de familiares/amigos de beneficiários	☐4	☐4
- Promover a doação por parte de pessoas envolvidas em tratamentos de fertilidade	☐5	☐5
- Alterar a legislação para que os dadores possam ser identificados	☐6	☐6

15.1. É importante, para nós, compreender melhor a sua resposta. **Explique-nos, por favor, a sua opinião.**

____

____

____

16. Neste momento, Portugal tem um Banco Público de Gâmetas no Porto e recolhem-se óvulos e espermatozoides em centros públicos situados em Coimbra e em Lisboa. **Em que medida concorda com esta localização dos centros públicos?**

Discordo totalmente	Discordo um pouco	Não concordo nem discordo	Concordo um pouco	Concordo totalmente
☐1	☐2	☐3	☐4	☐5

16.1. É importante, para nós, compreender melhor a sua resposta. **Explique-nos, por favor, a sua opinião.**

____

____

____

GRUPO II. PREDISPOSIÇÃO PARA DOAR E RECEBER GÂMETAS

17. Se tivesse todas as condições para poder doar gâmetas, em que medida estaria disponível para:

	Não disponível				Sempre disponível
- Doar gâmetas a familiares	☐0	☐1	☐2	☐3	☐4
- Doar gâmetas a amigos	☐0	☐1	☐2	☐3	☐4
- Doar gâmetas a desconhecidos	☐0	☐1	☐2	☐3	☐4
- Doar gâmetas para projetos de investigação científica	☐0	☐1	☐2	☐3	☐4

17.1. É importante, para nós, compreender melhor a sua resposta. **Explique-nos, por favor, as razões que justificam a sua posição.**

____

____

17.2. Em que medida estaria disponível para doar gâmetas para projetos de investigação científica:

	Não disponível				Sempre disponível
- Sem fins lucrativos	☐0	☐1	☐2	☐3	☐4
- Com fins lucrativos	☐0	☐1	☐2	☐3	☐4

17.3. Se os seus gâmetas fossem utilizados em projetos de investigação, gostaria de ser informado sobre isso?

- Sim, gostaria de ser sempre informado sobre o uso dos meus gâmetas ☐1
- Sim, gostaria de ser informado algumas vezes sobre o uso dos meus gâmetas ☐2
- Não gostaria de ser informado de cada vez que os meus gâmetas fossem utilizados ☐3
- Não sei ☐4

18. Aconselharia um familiar ou amigo próximo a fazer um tratamento com espermatozoides doados:

	**Não**	**Sim**	**Talvez**
- Por um irmão	☐0	☐1	☐2
- Por outro familiar próximo	☐0	☐1	☐2
- Por um amigo	☐0	☐1	☐2
- Por um desconhecido	☐0	☐1	☐2

19. Aconselharia uma familiar ou amiga próxima a fazer um tratamento com óvulos doados:

	**Não**	**Sim**	**Talvez**
- Por uma irmã	☐0	☐1	☐2
- Por outra familiar próxima	☐0	☐1	☐2
- Por uma amiga	☐0	☐1	☐2
- Por uma desconhecida	☐0	☐1	☐2

GRUPO III. PREDISPOSIÇÃO PARA DOAR EMBRIÕES

20. Imagine que seria beneficiário/a da doação de gâmetas e desse tratamento resultariam vários embriões "excedentários". **Em que medida estaria disponível para:**

	Não disponível				Sempre disponível
- Doar embriões para outros casais	☐0	☐1	☐2	☐3	☐4
- Doar embriões para projetos de investigação científica	☐0	☐1	☐2	☐3	☐4

21. Os embriões "excedentários" que resultam de tratamentos com doação de gâmetas podem ser doados para investigação científica. **Na sua opinião, quem deve autorizar ou recusar a doação destes embriões para <u>projetos de investigação</u>?**

- As pessoas que beneficiam do tratamento de fertilidade ☐1
- As pessoas que doaram os gâmetas ☐2
- Ambos (beneficiários e dadores) ☐3

22. Numa escala de 1 a 5, em que 1 é "Discordo totalmente" e 5 "Concordo totalmente", **em que medida concorda com as seguintes afirmações acerca da investigação na área da saúde?**

	Discordo totalmente	Discordo um pouco	Não concordo nem discordo	Concordo um pouco	Concordo totalmente
1. Tenho uma visão positiva sobre a investigação médica, em geral	☐1	☐2	☐3	☐4	☐5
2. A principal motivação dos investigadores na área da medicina é o benefício pessoal	☐1	☐2	☐3	☐4	☐5
3. Pode-se confiar nos investigadores para proteger os interesses das pessoas que participam nos seus estudos	☐1	☐2	☐3	☐4	☐5
4. Todos temos alguma responsabilidade em ajudar os outros ao voluntariarmo-nos para participar na investigação médica	☐1	☐2	☐3	☐4	☐5
5. A ciência moderna gera mais danos do que benefícios	☐1	☐2	☐3	☐4	☐5
6. A sociedade precisa de dedicar mais recursos à investigação médica	☐1	☐2	☐3	☐4	☐5
7. A investigação médica precisa de ser rigorosamente regulamentada de forma a prevenir danos nos participantes	☐1	☐2	☐3	☐4	☐5
8. Participar na investigação médica é, em geral, seguro	☐1	☐2	☐3	☐4	☐5
9. Se eu me voluntariar para a investigação médica, sei que a minha informação pessoal será mantida privada e confidencial	☐1	☐2	☐3	☐4	☐5
10. Dar muita ênfase à investigação médica e ao progresso científico pode prejudicar quem se voluntaria para a investigação	☐1	☐2	☐3	☐4	☐5
11. A investigação médica vai encontrar cura para muitas doenças importantes durante a minha vida	☐1	☐2	☐3	☐4	☐5

GRUPO IV. CARACTERÍSTICAS SOCIODEMOGRÁFICAS

23. Sexo:

Feminino	☐1
Masculino	☐2

24. Ano de nascimento: |___|___|___|___|

25. De onde é natural?

Portugal	☐1
Outro país	☐2

Qual?_________________________________

26. Onde reside atualmente? Distrito: ____

27. Qual é o seu estatuto marital?

Solteiro/a	☐1	Viúvo/a	☐4
Casado/a	☐2	Divorciado/a	☐5
União de facto	☐3	Separado/a (casado/a, mas não vive com o cônjuge)	☐6

28. Qual o grau de escolaridade mais elevado que completou?

Nenhum, e não sabe ler nem escrever	☐1	Ensino secundário (12.º ano)	☐6
Nenhum, mas sabe ler e escrever	☐2	Bacharelato	☐7
1.º Ciclo do ensino básico (4.º ano)	☐3	Licenciatura	☐8
2.º Ciclo do ensino básico (6.º ano)	☐4	Mestrado/Mestrado Integrado	☐9
3.º Ciclo do ensino básico (9.º ano)	☐5	Doutoramento	☐10

29. Neste momento, qual é a sua principal situação profissional? **(assinale apenas uma opção)**

Empregado/a a tempo inteiro	☐1	Reformado/a e pré-reformado/a	☐5
Empregado/a a tempo parcial	☐2	Doméstico/a /ocupa-se das tarefas do lar	☐6
Desempregado/a	☐3	Outra:	☐7
Estudante/ na escola/ em formação profissional	☐4	Qual?____________________________________	

30. Qual é a sua profissão atual? **(se na questão anterior assinalou desempregado/a, doméstico/a ou outra, p.f., considere a última profissão)**

____

____

31. Considera que os rendimentos do seu agregado familiar são:

Insuficientes	☐1
Tem de ter cuidado com os gastos	☐2
Chega para as suas necessidades	☐3
Confortáveis	☐4

32. Algumas pessoas consideram que a sociedade portuguesa está dividida em classes sociais. **Das seguintes classes, em qual delas se incluiria?**

Classe baixa	☐1
Classe média baixa	☐2
Classe média alta	☐3
Classe alta	☐4
Em nenhuma destas	☐5
Prefere não dizer	☐6

33. Tem filhos?

Não	☐0	**(p.f. passe para a pergunta 34)**
Sim	☐1	

33.1. Quantos filhos tem? ____________________

34. Alguma vez lhe foi diagnosticada infertilidade?

Não	☐0
Sim	☐1

Muito obrigada pela sua colaboração!
